# Antibacterial and Antifungal Properties of New Synthetic Tricyclic Flavonoids

**DOI:** 10.3390/antibiotics14030307

**Published:** 2025-03-16

**Authors:** Laura Gabriela Sarbu, Irina Rosca, Mihail Lucian Birsa

**Affiliations:** 1 Department of Chemistry, Alexandru Ioan Cuza University of Iasi, No. 11 Carol I Blvd., 700506 Iasi, Romania; 2 Intelcenter, Petru Poni Institute of Macromolecular Chemistry, No. 41A Grigore Ghica Voda Alley, 700487 Iasi, Romania; rosca.irina@icmpp.ro

**Keywords:** benzopyran, antibacterial, antifungal, synthetic flavonoids

## Abstract

**Background/Objectives:** The discovery of new molecules to which bacteria have not yet developed resistance is a significant medical priority. Synthetic flavonoids are good candidates for developing new antimicrobials. Our study investigates a series of newly synthesized tricyclic flavonoids with several different substituents on the flavonoid core. **Methods:** By varying the nature of the substituents on rings **A** and **B,** a structure–activity relationship study using different microbial strains has been performed. The antibacterial and antifungal properties of these compounds have been investigated against Gram-positive and Gram-negative bacteria and several *Candida* strains. **Results:** All seven tested compounds have been found to exhibit the highest antimicrobial activity against *S. aureus*, with an inhibition zone of up to 23 mm. The tricyclic flavonoids **5c**, **5e**, and **5f** showed good antifungal properties against *C. parapsilosis*, with an inhibition zone of around 17 mm. **Conclusions:** All the data support the idea that flavonoids **5** are reliable candidates for developing effective antimicrobial agents.

## 1. Introduction

Regarded by the World Health Organization (WHO) as one of the top issues in public health worldwide, antimicrobial resistance (AMR) is estimated to have been associated with 4.95 million deaths in 2019 alone [1]. The inappropriate use of antibiotics in both medicine and agriculture represents one of the main causes of AMR. Given the current trend, if no steps are taken to address the issue, AMR is projected to lead to as many as 10 million deaths per year, globally, by 2050 [2]. Apart from its impact on human health, AMR has significant implications from a socioeconomic point of view, with an estimated loss in global gross domestic product of 3.8% by the year 2050 [3].

Microorganisms that have developed resistance to antibiotics have no problem surviving or even multiplying, despite the administration of antibiotics [4]. This leads to a loss in effectiveness when it comes to antimicrobials, which makes it increasingly difficult—and indeed, sometimes impossible—to treat certain infections, further adding pressure to already strained healthcare systems globally [5]. On top of that, the problem of AMR is further compounded by the emergence of multidrug-resistant (MDR) microorganisms, which are responsible for an increase in the incidence of nosocomial infections [6]. There are several limitations and challenges in the development of new antibiotics and antifungals as well as regulatory processes that impede advancement in this critical field. The degree of resistance expression of the bacterial strain and its capacity to survive through resistance mechanisms are two of the many elements that contribute to the expression of antibiotic resistance against drug agents [7]. Due to natural processes, first-generation antibiotics have been affected by antibiotic resistance in a variety of therapeutic applications [8].

Although there has been some progress in the fight against antimicrobial resistance, β-lactamic antibiotics and related compounds are not a viable solution against MDR strains anymore. Referring to a recent WHO report, in the last seven years, only thirteen new antibiotics have been authorized and only two (vaborbactam and lefamulin) represent new classes of antibiotics [9]. Furthermore, the newly approved drugs are not more effective and do not exhibit new mechanisms of action against the present threats.

Among the many problematic microbes, *Candida* pathogenic species have a significant impact on public health [10]. With a mortality rate of 40–60%, particularly in immunocompromised patients, *C. albicans* is the most widely encountered pathogen in the *Candida* genus [11]. *Candida* infections can be treated using antifungals such as azoles, allylamines, echinocandins and polyenes, as well as nucleoside analogs [12]. The effectiveness of antifungal drugs is, however, challenged by multidrug resistance. This is well documented for *Candida* species, particularly in the case of azoles [10]. It was found that one of the main causes of MDR in *Candida* spp. is its ability to form biofilms [13]. Within biofilms, it was found that cells can withstand antifungal concentrations up to 10^3^ larger than planktonic cells [14]. The lack of effective treatments poses a significant challenge for modern medicine when it comes to *Candida* infections.

In this context, the need for innovative approaches in developing novel antimicrobials efficient in treating infectious diseases is a major goal.

From this point of view, flavonoids—a large group of naturally occurring heterocyclic organic compounds found in fruits, vegetable, tea, and wine—are potentially good candidates as antimicrobial agents. Associated with the multitude of substitution patterns on the C_6_-C_3_-C_6_ backbone, more than 12,000 flavonoids are known. The attention that they receive is a direct consequence of the many biological activities that this class of compounds displays. Flavonoids have a wide variety of chemical and biochemical properties that provide them antimicrobial properties [15,16,17]. These compounds are also known to be good antioxidants [18,19,20,21,22,23]. Moreover, certain studies suggest that flavonoids could offer protection against cancer, as well as cardiovascular diseases [24,25,26,27,28,29,30,31,32].

**Figure 1:**
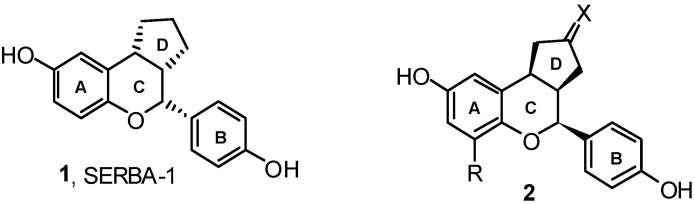
Polycyclic flavonoids.

Benzopyran derivatives, like SERBAs or selective estrogen receptor β agonists, are compounds that interact with estrogen receptor subtypes α and β. The interest for this type of compound leads to derivative **1**, a selective estrogen receptor β agonist (SERBA-1, Figure 1) [33]. Studies about the structure–activity relationship have been presented [34,35]. It has been shown that a cyclopenthane ring at the 3,4-carbon positions (labeled **D**, Figure 1) leads to a substantial rise in binding affinity for estrogen receptor β. By combining improvements in the **D** ring with those in the **A** ring, improvements in binding selectivity have been obtained (e.g., **2**, Figure 1) [36].

Regardless of their potential, the use of natural flavonoids in medicine is hindered by issues such as limited bioavailability, poor solubility, and stability [37]. As such, new flavonoids with potent antimicrobial properties designed to address the aforementioned issues have been synthesized. Over the past twelve years, we developed a novel type of tricyclic flavonoid containing a 1,3-dithiolium ring (similar to **D** in Figure 1) and featuring various substituents on the C_6_-C_3_-C_6_ backbone. We effectively demonstrated that these synthetic flavonoids possess strong antibacterial and antifungal abilities and examined how they function [38,39,40].

We are reporting here an extension of our studies about the development of new tricyclic flavonoids with good antimicrobial activities against several microbial strains. By varying the nature of the substituents on the rings **A** and **B,** we intended to perform a study on the structure–activity relationship using different microbial strains.

## 2. Results and Discussion

### 2.1. Tricyclic Flavonoids

**Scheme 1:**
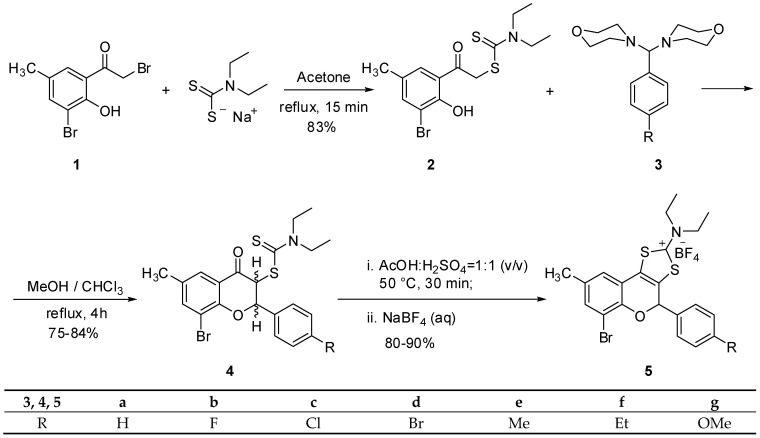
Tricyclic flavonoids **5a**–**g**.

The synthesis of 4-chromanone and flavanone derivatives has been previously achieved through the interaction of type **2** dithiocarbamates with aminals **3** [41]. Based on our prior research, we aimed to broaden our investigations to include other new tricyclic flavonoids similar in structure to those illustrated in Figure 1. As a result, 3-dithiocarbamic flavanones **4** have been obtained by reacting 1-(3-bromo-2-hydroxy-5-methylphenyl)-1-oxaethan-2-yl *N,N*-diethylaminocarbodithioate (**2**) with aminals **3** (Scheme 1). The preparation of the latter compounds followed established methods described in the literature [42,43]. Thus, by reacting 2-bromo-1-(3-bromo-2-hydroxy-5-methylphenyl)ethan-1-one (**1**) [44] with sodium *N,N*-diethyldithiocarbamate in acetone, phenacyl aminocarbodithioate **2** has been successfully synthesized in good yields. The reaction of 1-(3-bromo-2-hydroxy-5-methylphenyl)-1-oxaethan-2-yl *N,N*-diethylaminocarbodithioate (**2**) with aminals **3** resulted in the formation of 3-substituted dithiocarbamic flavanones **4a**–**g** as a mixture of diastereoisomers (Scheme 1). The reaction mixture underwent reflux heating for 3.5 h, and following recrystallization from ethanol, 3-dithiocarbamic flavanones **4a**–**g** were obtained, with yields ranging from 75% to 84%. NMR spectroscopy clearly shows the ring closure of the benzopyran core. In addition to the NMR pattern of the newly para-substituted aromatic ring B, the loss of the methylene group’s signal from dithiocarbamate **2** (4.79 ppm) and the presence of the distinctive signals of vicinal hydrogen atoms at the C-2 and C-3 positions of the benzopyran ring for both diastereoisomers are observed between 5.8 and 6.3 ppm. Mass spectra also supported the ring closure to the benzopyrane ring.

**Figure 2:**
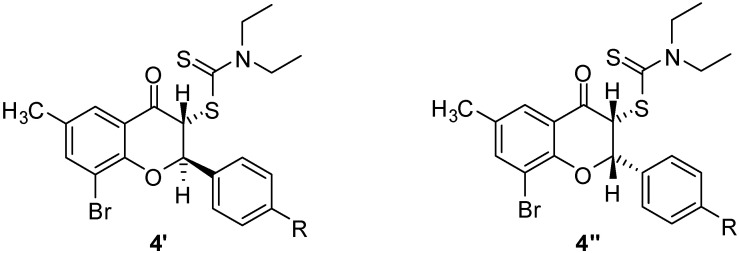
Diastereoisomers of flavanones **4**.

Based on the stereochemical principles, these flavanones should have two diastereoisomers that originate from the relative orientation of H-2 and H-3 hydrogen atoms of the flavanones **4,** which can be directed either to opposite sides or to the same side of the benzopyrane core (Figure 2). The relative orientations of the two substituents induce an influence on the magnitude of their coupling constants. It is reasonable to assume that the most stable isomer should be the *anti* diastereomer **4**′, as opposed to the *syn* diastereomer **4**″. However, it has been found that in the case of two flavanones, **4b** and **4g**, the *syn* isomer has been the major one. The diastereoisomeric ratio and coupling constants of flavanones **4** are presented in Table 1.

Phenacyl dithiocarbamates are valuable precursors for 2-dialkylamino-1,3-dithiolium-2-yl cations [45,46]. Cyclocondensation under acidic conditions of these substrates is the method employed for the preparation of 1,3-dithiolium cations [47]. The cyclization of 3-dithiocarbamic flavanones **4** to tricyclic flavonoids **5** has been proven by spectral data.

IR spectroscopy indicated the disappearance of the carbonyl absorption bands (1690–1705 cm^−1^) and the appearance of the characteristic broad absorption bands (ca. 1080–1095 cm^−1^) of the tetrafluoroborate anion. The ^1^H NMR spectra of tricyclic flavonoids **5** show the disappearance of the C-3 hydrogens from the benzopyran core. Furthermore, the signals of the C-2 hydrogens have been shifted to ca. 6.8 ppm, where they can be found as singlets. The ^13^C NMR spectra indicate the absence of the carbonyl and thiocarbonyl atoms and present a new signal at ca. 184 ppm, corresponding to the 1,3-dithiol-2-ylium carbon atom.

### 2.2. Antimicrobial Activity of Tricyclic Flavonoids

**Figure 3:**
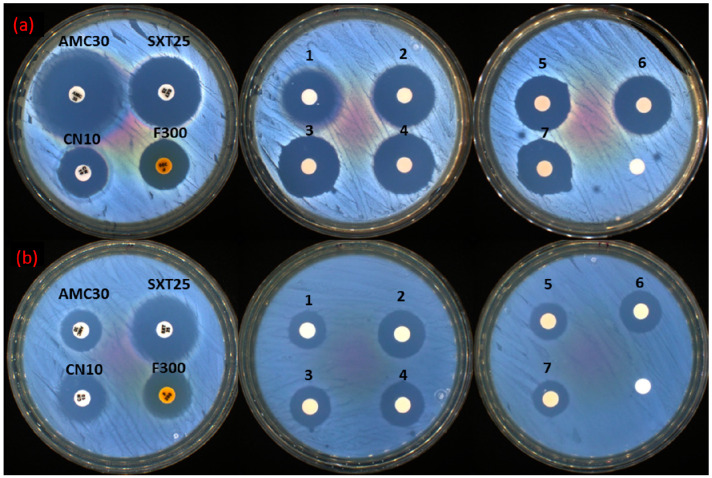
Antibacterial activity of tested controls (**AMC30**—amoxyclav, **SXT25**—co-trimoxazole, **CN10**—gentamicin, **F300**—nitrofurantoin) and samples (1—**5a**, 2—**5b**, 3—**5c**, 4—**5e**, 5—**5f**, 6—**5g**, 7—**5d**) against (**a**)—*S. aureus*, (**b**)—*K. pneumoniae*.

Almost all the tested flavonoids exhibited both antibacterial and antifungal activity against the tested microbial strains, as presented in Table 2a. Clearly, the flavonoids were more efficient against the bacterial strains, especially against the Gram-positive bacterial strain represented by *S. aureus*, with an inhibition zone of up to 23 mm in the particular cases of tricyclic flavonoids **5b**, **5c**, **5e**, and **5g** (See Figure 3a). Moderate efficiency was noticed against *E. faecalis* (with an inhibition zone of up to 15 mm in the particular case of **5e**) (as presented in Figure 3b) and *E. coli* (with an inhibition zone of up to 15 mm in the case of **5c**). Flavonoid **5a** was quite efficient against *S. aureus* but was not efficient against the other bacterial strains. The same flavonoid exhibited very low activity against *C. parapsilosis* but did not present activity against the other yeast strains. None of the flavonoids were efficient against the Gram-negative bacterial strain represented by *P. aeruginosa*; however, all of the tested flavonoids were quite efficient against *C. parapsilosis*, withan inhibition zone of up to 17 mm in the case of flavonoid **5e**. The antifungal activity of **5e** against the other strains was very low for the two strains of *C. albicans* and the two strains of *C. glabrata*.

When compared with the commercial controls, it was noticed that flavonoid **5a** had the same efficiency as **CN10** when tested against *S. aureus* (as presented in Table 2b, Figure 3a). Flavonoids **5b**–**5g** had higher activity than **CN10** and **F300** against the same bacterial strain. In the case of *E. coli*, none of the flavonoids were more efficient than the controls. For *E. faecalis*, only flavonoid **5e** had higher efficiency than **CN10**. Flavonoids **5f** and **5g** had the same activity as **AMC30** against *K. pneumoniae*, as presented in Table 2a,b, Figure 3b.

In the case of antifungals, **AP100** had the same efficiency as flavonoids **5c**,**d** against *C. albicans* ATCC 90028, while **5e** was better than **KCA10**. In addition, for this particular strain, **5f** had better efficiency than all the tested controls. For the yeast strain *C. albicans* ATCC 10231, control **AP100** had the same activity as **5d**, and all the flavonoids were more efficient than the rest of the tested controls (**NS100**, **KCA10,** and **FLU10**). In the case of *C. glabrata* ATCC 15126, control sample **AP100** was more efficient than all the tested flavonoids; however, flavonoids **5c**–**f** were better than **NS100**, **KCA10**, and **FLU10**. A similar situation was noticed in the case of *C. glabrata* ATCC 20019, when comparing the flavonoids’ activity with control **AP100**; however, **5f** was better than **NS100**, **KCA10,** and **FLU10**. For *C. parapsilosis*, flavonoids **5a** and **5g** had reduced efficiency when compared to **AP100**, while the remaining flavonoids were more efficient than the same control. Furthermore, for this yeast strain, **5d** had the same activity as **FLU10**, while flavonoids **5c**, **5e** and **5f** were better than this control. At the same time, all tested flavonoids exhibited better activity than **NS100** and **KCA10**.

## 3. Materials and Methods

Melting points were obtained on a *KSPI* melting-point meter (A. KRÜSS Optronic, Hamburg, Germany) and are uncorrected. IR spectra were recorded on a Bruker Tensor 27 instrument (Bruker Optik GmbH, Ettlingen, Germany). NMR spectra were recorded on a Bruker 500 MHz spectrometer (Bruker BioSpin, Rheinstetten, Germany). Chemical shifts are reported in ppm downfield from TMS. Mass spectra were recorded on a Thermo Scientific ISQ LT instrument (Thermo Fisher Scientific Inc., Waltham, MA, USA). All reagents were commercially available and used without further purification. Elemental analysis and copies of ^13^C-NMR spectra are available in the Supplementary Materials.

The controls for the antimicrobial tests included the following: amoxicillin/clavulanic acid, 20/10 µg/disc (AMC30); co-trimoxazole 25 µg/disc (SXT25); gentamicin, 10 µg/disc (CN10), nitrofurantoin, 300 µg/disc (F300), from Tody Laboratories (Bucharest, Romania); amphotericin B, 100 µg/disc (AP100) from HiMedia Laboratories (Mumbai, India); nystatin, 100 µg/disc (NS100); ketoconazole, 10 µg/disc (KCA10) from Tody Laboratories (Bucharest, Romania); and fluconazole, 10 µg/disc (FLU10) from Bioanalyse Limited (Ankara, Turkey).

### 3.1. Chemistry

#### 3.1.1. General Procedure for 8-Bromo-6-methyl-2-phenyl-4-oxochroman-3-yl N,N-Diethyldithiocarbamate (**4a**)

To a solution of 1-(5-methyl-2-hydroxyphenyl)-1-oxoethan-2-yl *N,N*-diethyldithiocarbamate (**2**) (0.376 g, 1 mmol) in a mixture of CHCl_3_/CH_3_OH (15 mL, 1:1 *v*/*v*), aminal **3a** (0.262 g, 1 mmol) was added and the reaction mixture was heated under reflux for 3.5 h. After cooling, the solid material was filtered off and was recrystallized from ethanol to give **4a** (0.39 g, 81%) as colorless crystals. IR (ATR, cm^−1^) 2981, 1693, 1492, 1465, 1202, 976, 766, 694, 562. ^1^H NMR (CDCl_3_, selected data for the major *anti* isomer) δ 7.63 (m, 2H), 7.52 (m, 2H), 7.35 (m, 3H), 6.00 (d, *J* = 6.9 Hz, 1H), 5.82 (d, *J* = 6.9 Hz, 1H), 3.94 (m, 2H), 3.67 (m, 2H), 2.30 (s, 3H), 1.24 (m, 6H). ^13^C NMR (CDCl_3_, selected data for the major *anti* isomer) δ 191.5, 187.4, 154.8, 140.5, 136.3, 132.3, 128.6, 128.5, 127.2, 126.7, 122.0, 111.5, 82.8, 58.5, 50.5, 47.2, 20.2, 12.6, 11.4. MS (EI) *m/z*: 463.1 (M^+^, 24%) for C_21_H_22_^79^BrNO_2_S_2_.

#### 3.1.2. 8-Bromo-6-methyl-2-(4-fluorophenyl)-4-oxochroman-3-yl N,N-Diethyldithiocarbamate (**4b**)

White solid, 0.35 g, 78%. IR (ATR, cm^−1^) 2989, 1694, 1493, 1469, 1269, 1198, 808, 555. ^1^H NMR (CDCl_3_, selected data for the major *syn* isomer) δ 7.72 (d, *J* = 2.0 Hz, 1H), 7.61 (d, *J* = 2.0 Hz, 1H), 7.51 (m, 2H), 7.05 (m, 2H), 6.21 (d, *J* = 3.8 Hz, 1H), 5.98 (d, *J* = 3.8 Hz, 1H), 3.98 (m, 2H), 3.67 (m, 2H), 2.33 (s, 3H), 1.21 (m, 6H). ^13^C NMR (CDCl_3_, selected data for the major *syn* isomer) δ 191.0, 188.0, 163.7, 154.5, 140.4, 132.3, 131.2, 129.3, 128.8, 126.8, 121.2, 115.3, 111.6, 80.6, 57.9, 51.0, 47.0, 20.2, 12.6, 11.3. MS (EI) *m/z*: 481.1 (M^+^, 19%) for C_21_H_21_^79^BrFNO_2_S_2_.

#### 3.1.3. 8-Bromo-6-methyl-2-(4-chlorophenyl)-4-oxochroman-3-yl N,N-Diethyldithiocarbamate (**4c**)

Pale yellow solid, 0.34 g, 79%. IR (ATR, cm^−1^) 2972, 1692, 1492, 1470, 1271, 1200, 803, 549. ^1^H NMR (CDCl_3_, selected data for the major *anti* isomer) δ 7.66 (m, 1H), 7.64 (m, 1H), 7.48 (d, *J* = 8.5 Hz, 2H), 7.33 (d, *J* = 8.5 Hz, 2H), 5.93 (d, *J* = 7.1 Hz, 1H), 5.80 (d, *J* = 7.1 Hz, 1H), 3.93 (m, 2H), 3.70 (m, 2H), 2.32 (s, 3H), 1.25 (m, 6H). ^13^C NMR (CDCl_3_, selected data for the major *anti* isomer) δ 191.3, 187.1, 154.7, 140.6, 134.6, 133.9, 132.6, 128.8, 128.6, 126.8, 121.9, 111.4, 82.3, 58.7, 50.6, 47.2, 20.2, 12.6, 11.3. MS (EI) *m/z*: 497.0 (M^+^, 14%) for C_21_H_21_^79^BrClNO_2_S_2_.

#### 3.1.4. 8-Bromo-6-methyl-2-(4-bromophenyl)-4-oxochroman-3-yl N,N-Diethyldithiocarbamate (**4d**)

White solid, 0.37 g, 84%. IR (ATR, cm^−1^) 2977, 1691, 1489, 1469, 1271, 1200, 801, 549. ^1^H NMR (CDCl_3_, selected data for the major *anti* isomer) δ 7.65 (d, *J* = 1.8 Hz, 1H), 7.63 (d, *J* = 1.8 Hz, 1H), 7.48 (d, *J* = 8.5 Hz, 2H), 7.42 (d, *J* = 8.5 Hz, 1H), 5.91 (d, *J* = 7.2 Hz, 1H), 5.79 (d, *J* = 7.2 Hz, 1H), 3.93 (m, 2H), 3.70 (m, 2H), 2.32 (s, 3H), 1.24 (m, 6H). ^13^C NMR (CDCl_3_, selected data for the major *anti* isomer) δ 191.3, 187.1, 154.7, 140.6, 135.3, 132.6, 131.6, 129.1, 126.8, 122.9, 121.9, 111.4, 82.3, 58.7, 50.6, 47.3, 20.2, 12.6, 11.4. MS (EI) *m/z*: 540.9 (M^+^, 14%) for C_21_H_21_^79^Br_2_NO_2_S_2_.

#### 3.1.5. 8-Bromo-6-methyl-2-(4-methylphenyl)-4-oxochroman-3-yl N,N-Diethyldithiocarbamate (**4e**)

Pale yellow solid, 0.31 g, 77%. IR (ATR, cm^−1^) 2981, 1695, 1494, 1468, 1269, 1202, 798, 547. ^1^H NMR (CDCl_3_, selected data for the major *anti* isomer) δ 7.63 (m, 1H), 7.62 (m, 1H), 7.41 (d, *J* = 8.0 Hz, 2H), 7.16 (d, *J* = 8.0 Hz, 2H), 5.96 (d, *J* = 7.6 Hz, 1H), 5.79 (d, *J* = 7.6 Hz, 1H), 3.94 (m, 2H), 3.70 (m, 2H), 2.33 (s, 3H), 2.30 (s, 3H), 1.24 (m, 6H). ^13^C NMR (CDCl_3_, selected data for the major *anti* isomer) δ 191.6, 187.5, 154.8, 140.5, 138.5, 133.3, 132.2, 129.2, 127.1, 126.7, 122.0, 111.5, 82.7, 58.5, 50.4, 47.2, 21.2, 20.2, 12.6, 11.4. MS (EI) *m/z*: 477.1 (M^+^, 21%) for C_22_H_24_^79^BrNO_2_S_2_.

#### 3.1.6. 8-Bromo-6-methyl-2-(4-ethylphenyl)-4-oxochroman-3-yl N,N-Diethyldithiocarbamate (**4f**)

White solid, 0.3 g, 75%. IR (ATR, cm^−1^) 2978, 1691, 1491, 1416, 1261, 1236, 796, 553. ^1^H NMR (CDCl_3_, selected data for the major *anti* isomer) δ 7.64 (m, 1H), 7.62 (m, 1H), 7.43 (d, *J* = 8.1 Hz, 2H), 7.18 (d, *J* = 8.1 Hz, 2H), 5.98 (m, 1H), 5.79 (m, 1H), 3.95 (m, 2H), 3.70 (m, 2H), 2.63 (q, *J* = 7.6 Hz, 2H), 2.31 (s, 3H), 1.24 (m, 6H), 1.22 (t, *J* = 7.6 Hz, 3H). ^13^C NMR (CDCl_3_, selected data for the major *anti* isomer) δ 191.6, 187.5, 154.9, 144.7, 140.5, 133.5, 132.2, 128.0, 127.2, 126.7, 122.0, 111.5, 82.7, 58.4, 50.4, 47.2, 28.5, 20.2, 15.3, 12.6, 11.4. MS (EI) *m/z*: 491.1 (M^+^, 16%) for C_23_H_26_^79^BrNO_2_S_2_.

#### 3.1.7. 8-Bromo-6-methyl-2-(4-methoxyphenyl)-4-oxochroman-3-yl N,N-Diethyldithiocarbamate (**4g**)

White solid, 0.29 g, 79%. IR (ATR, cm^−1^) 2967, 1692, 1493, 1416, 1265, 1240, 1034, 817, 546. ^1^H NMR (CDCl_3_, selected data for the major *syn* isomer) δ 7.71 (m, 1H), 7.58 (m, 1H), 7.41 (d, *J* = 8.7 Hz, 2H), 6.88 (d, *J* = 8.7 Hz, 2H), 6.27 (d, *J* = 4.2 Hz, 1H), 5.97 (d, *J* = 4.2 Hz, 1H), 3.95 (m, 2H), 3.78 (s, 3H), 3.72 (m, 2H), 2.31 (s, 3H), 1.23 (m, 6H). ^13^C NMR (CDCl_3_, selected data for the major *syn* isomer) δ 191.5, 188.1, 159.7, 154.5, 140.3, 132.3, 128.5, 127.5, 126.7, 121.4, 113.7, 111.6, 81.1, 58.4, 55.2, 50.8, 47.1, 20.2, 12.6, 11.4. MS (EI) *m/z*: 493.0 (M^+^, 21%) for C_22_H_24_^79^BrNO_3_S_2_.

#### 3.1.8. General Procedure for 2-N,N-Diethylamino-8-bromo-6-methyl-4-phenyl-4H-1,3-dithiol[4,5-c]chromen-2-ylium Tetrafluoroborate (**5a**)

To a mixture of sulfuric acid (1 mL) and acetic acid (1 mL), flavanone **4a** (0.232 g, 0.5 mmol) was added and the resulting solution was heated to 50 °C for 30 min. The reaction mixture was then left to cool to room temperature and a solution of sodium tetrafluoroborate (0.3 g) in water (10 mL) was added dropwise, under vigorous stirring. The resulting precipitate was then filtered, washed thoroughly with water, and recrystallized from ethanol, yielding the desired tetrafluoroborate **5a** in the form of colorless crystals (0.22 g, 88%). m.p. 249–250 °C. IR (ATR, cm^−1^) 1540, 1444, 1041, 710, 421. ^1^H NMR (DMSO-*d*_6_) δ 7.47 (m, 5H), 7.27 (s, 1H), 7.18 (d, *J* = 8.2 Hz, 1H), 6.95 (d, *J* = 8.2 Hz, 1H), 6.71 (s, 1H), 3.93 (m, 2H), 3.84 (m, 2H), 2.28 (s, 3H), 1.39 (t, *J* = 7.1 Hz, 3H), 1.31 (t, *J* = 7.1 Hz, 3H). ^13^C NMR (DMSO-*d*_6_) δ 184.9, 149.3, 137.4, 132.9, 132.7, 130.3, 129.5, 128.5, 127.9, 127.6, 125.4, 117.5, 116.6, 75.5, 54.7, 54.5, 20.4, 10.8, 10.6. MS (EI) *m/z*: 446.0 (M^+^-BF_4,_ 5%) for C_21_H_21_^79^BrNOS_2_]^+^.

#### 3.1.9. 2-N,N-Diethylamino-8-bromo-6-methyl-4-(4-fluorophenyl)-4H-1,3-dithiol[4,5-c]chromen-2-ylium Tetrafluoroborate (**5b**)

White solid, m.p. 201–202 °C (0.21 g, 82%). IR (ATR, cm^−1^) 1559, 1464, 1224, 1051, 763, 561, 518. ^1^H NMR (DMSO-*d*_6_) δ 7.55 (m, 2H), 7.49 (m, 1H), 7.31 (m, 2H), 7.28 (m, 1H), 6.91 (s, 1H), 3.94 (m, 2H), 3.88 (m, 2H), 2.27 (s, 3H), 1.41 (t, *J* = 7.0 Hz, 3H), 1.34 (t, *J* = 7.0 Hz, 3H). ^13^C NMR (DMSO-*d*_6_) δ 185.0, 164.2, 145.7, 135.6, 134.5, 133.2, 130.3, 128.3, 128.2, 125.0, 117.9, 116.6, 110.8, 75.2, 54.7, 54.5, 20.1, 10.8, 10.6. MS (EI) *m/z*: 463.9 (M^+^-BF_4,_ 7%) for C_21_H_20_^79^BrFNOS_2_]^+^.

#### 3.1.10. 2-N,N-Diethylamino-8-bromo-6-methyl-4-(4-chlorophenyl)-4H-1,3-dithiol[4,5-c]chromen-2-ylium Tetrafluoroborate (**5c**)

White solid, m.p. 221–222 °C (0.26 g, 86%). IR (ATR, cm^−1^) 1549, 1455, 1273, 1044, 783, 519. ^1^H NMR (DMSO-*d*_6_) δ 7.52 (m, 4H), 7.49 (m, 1H), 7.31 (m, 1H), 6.93 (s, 1H), 3.94 (m, 2H), 3.89 (m, 2H), 2.27 (s, 3H), 1.41 (t, *J* = 7.1 Hz, 3H), 1.34 (t, *J* = 7.1 Hz, 3H). ^13^C NMR (DMSO-*d*_6_) δ 185.1, 145.7, 135.9, 135.6, 135.1, 134.6, 129.7, 129.6, 128.3, 128.0, 125.1, 117.9, 110.8, 75.1, 54.7, 54.6, 20.1, 10.8, 10.6. MS (EI) *m/z*: 480.0 (M^+^-BF_4,_ 8%) for C_21_H_20_^79^BrClNOS_2_]^+^.

#### 3.1.11. 2-N,N-Diethylamino-8-bromo-6-methyl-4-(4-bromophenyl)-4H-1,3-dithiol[4,5-c]chromen-2-ylium Tetrafluoroborate (**5d**)

White solid, m.p. 235–237 °C (0.23 g, 90%). IR (ATR, cm^−1^) 1548, 1477, 1282, 1029, 781, 519. ^1^H NMR (DMSO-*d*_6_) δ 7.66 (d, *J* = 8.4 Hz, 2H), 7.49 (m, 1H), 7.45 (d, *J* = 8.4 Hz, 2H), 7.31 (m, 1H), 6.91 (s, 1H), 3.94 (m, 2H), 3.89 (m, 2H), 2.27 (s, 3H), 1.41 (t, *J* = 7.1 Hz, 3H), 1.34 (t, *J* = 7.1 Hz, 3H). ^13^C NMR (DMSO-*d*_6_) δ 185.1, 145.7, 136.3, 135.6, 134.6, 132.5, 129.9, 128.3, 127.9, 125.1, 123.8, 117.9, 110.8, 75.2, 54.7, 54.5, 20.1, 10.8, 10.6. MS (EI) *m/z*: 523.8 (M^+^-BF_4,_ 4%) for C_21_H_20_^79^Br_2_NOS_2_]^+^.

#### 3.1.12. 2-N,N-Diethylamino-8-bromo-6-methyl-4-(4-methylphenyl)-4H-1,3-dithiol[4,5-c]chromen-2-ylium Tetrafluoroborate (**5e**)

White solid, m.p. 245–246 °C (0.19 g, 82%). IR (ATR, cm^−1^) 1548, 1449, 1283, 1045, 788, 560. ^1^H NMR (DMSO-*d*_6_) δ 7.48 (m, 1H), 7.38 (d, *J* = 7.8 Hz, 2H), 7.30 (m, 1H), 7.26 (d, *J* = 7.8 Hz, 2H), 6.84 (s, 1H), 3.93 (m, 2H), 3.87 (m, 2H), 2.31 (s, 3H), 2.27 (s, 3H), 1.40 (t, *J* = 7.1 Hz, 3H), 1.33 (t, *J* = 7.1 Hz, 3H). ^13^C NMR (DMSO-*d*_6_) δ 185.1, 146.1, 140.1, 135.5, 134.3, 134.0, 130.0, 128.8, 127.9, 127.8, 125.0, 118.0, 110.8, 75.9, 54.7, 54.5, 21.2, 20.1, 10.8, 10.6. MS (EI) *m/z*: 460.0 (M^+^-BF_4,_ 9%) for C_22_H_23_^79^BrNOS_2_]^+^.

#### 3.1.13. 2-N,N-Diethylamino-8-bromo-6-methyl-4-(4-ethylphenyl)-4H-1,3-dithiol[4,5-c]chromen-2-ylium Tetrafluoroborate (**5f**)

White solid, m.p. 205–207 °C (0.18 g, 83%). IR (ATR, cm^−1^) 1548, 1449, 1283, 1044, 785, 518. ^1^H NMR (DMSO-*d*_6_) δ 7.49 (m, 1H), 7.40 (d, *J* = 8.0 Hz, 2H), 7.30 (d, *J* = 8.0 Hz, 2H), 7.28 (m, 1H), 6.86 (s, 1H), 3.93 (m, 2H), 3.87 (m, 2H), 2.61 (q, *J* = 7.5 Hz, 2H), 2.27 (s, 3H), 1.40 (t, *J* = 7.1 Hz, 3H), 1.33 (t, *J* = 7.1 Hz, 3H), 1.17 (t, *J* = 7.6 Hz, 3H). ^13^C NMR (DMSO-*d*_6_) δ 185.0, 146.2, 146.0, 135.5, 134.4, 134.3, 128.9, 128.7, 128.0, 127.9, 125.0, 118.0, 110.8, 75.9, 54.7, 54.5, 28.3, 20.1,15.8, 10.8, 10.6. MS (EI) *m/z*: 474.1 (M^+^-BF_4,_ 4%) for C_23_H_25_^79^BrNOS_2_]^+^.

#### 3.1.14. 2-N,N-Diethylamino-8-bromo-6-methyl-4-(4-methoxyphenyl)-4H-1,3-dithiol[4,5-c]chromen-2-ylium Tetrafluoroborate (**5g**)

White solid, m.p. 219–220 °C (0.14 g, 83%). IR (ATR, cm^−1^) 1542, 1456, 1254, 1179, 1051, 792, 519. ^1^H NMR (DMSO-*d*_6_) δ 7.48 (m, 1H), 7.42 (d, *J* = 8.6 Hz, 2H), 7.30 (m, 1H), 7.00 (d, *J* = 8.6 Hz, 2H), 6.82 (s, 1H), 3.93 (m, 2H), 3.87 (m, 2H), 3.77 (s, 3H), 2.27 (s, 3H), 1.40 (t, *J* = 7.1 Hz, 3H), 1.32 (t, *J* = 7.1 Hz, 3H). ^13^C NMR (DMSO-*d*_6_) δ 184.9, 160.8, 146.1, 135.5, 134.2, 129.6, 129.1, 128.8, 127.9, 125.0, 118.0, 114.8, 110.8, 75.8, 55.7, 54.7, 54.5, 20.1, 10.8, 10.6. MS (EI) *m/z*: 476.0 (M^+^-BF_4,_ 6%) for C_22_H_23_^79^BrNO_2_S_2_]^+^.

### 3.2. Antimicrobial Activity

The antimicrobial activity of the samples was determined by the disk diffusion assay method [48,49] against 10 microbial reference Gram-positive bacterial strains represented by *Staphylococcus aureus* ATCC 25923 and *Enterococcus faecalis* ATCC 29212. The Gram-negative bacterial strains were represented by *Escherichia coli* ATCC 25922, *Klebsiella pneumoniae* ATCC 10031, and *Salmonella typhimurium* ATCC 14028, and the yeast strains were represented by *Candida albicans* ATCC 90028, *Candida albicans* ATCC 10231, *Candida glabrata* ATCC 20019, *Candida glabrata* ATCC 15126, and *Candida parapsilosis* ATCC 22019. All microorganisms were stored at −80 °C in 20% glycerol. The bacterial strains were refreshed on nutrient agar (NA) at 37 °C, and the yeast strains were refreshed on Sabouraud dextrose agar (SDA). Microbial suspensions were prepared with these cultures in a sterile solution to obtain turbidity optically comparable to that of 0.5 McFarland standards. Volumes of 0.1 mL from each inoculum were spread onto NA/SDA plates, and 15 µL of the samples (concentration 3 mg/mL) were added. To evaluate the antimicrobial properties, the growth inhibition was measured under standard conditions after 24 h of incubation at 37 °C. All tests were carried out in triplicate to verify the results. Standard controls were tested in the same conditions. After incubation, the samples were analyzed with SCAN1200^®^, version 8.6.10.0 (Interscience, Saint Nom la Bretêche, France) and were expressed as the mean ± standard deviation (SD) performed with GraphPad Prism software version 7.00 for Windows (GraphPad Software, La Jolla, CA, USA, www.graphpad.com).

## 4. Conclusions

Our study focused its investigation on the synthesis and antimicrobial properties of a series of novel tricyclic flavonoids. By changing the nature of the substituents on the flavonoid rings **A** and **B,** a structure–activity relationship study using different microbial strains was performed. The antibacterial and antifungal properties of these compounds have been investigated against Gram-positive and Gram-negative bacteria and several *Candida* strains. All seven tested compounds were found to exhibit the highest antimicrobial activity against *S. aureus*, with an inhibition zone of around 20 mm for flavonoids **5a**, **5d**, **5f** and around 23 mm for **5b**, **5c**, **5e**, and **5g**. The tricyclic flavonoids **5c**, **5e**, and **5f** showed good antifungal properties against *C. parapsilosis*, with an inhibition zone of around 17 mm. Among these compounds, the tricyclic flavonoid **5e** with an ethyl substituent at the ring B exhibited the highest activity against both *S. aureus* and *C. parapsilosis* strains, with an inhibition zone of 23.03 and 17.43 mm, respectively. These results support the idea that the tricyclic flavonoids **5** are reliable candidates for developing effective antimicrobial agents.

## Figures and Tables

**Table 1:** Coupling constants and diastereoisomers ratio of flavanones **4**.

Flavanones 4	a	b	c	d	e	f	g
^3^*J*_H2-H3_*syn* (Hz)	3.9	3.8	3.7	3.8	4.2	3.9	4.2
^3^*J*_H2-H3_ *anti* (Hz)	6.9	7.4	7.1	7.2	7.6	7.2	7.4
*syn: anti* ratio	7:93	80:20	36:64	39:61	40:60	6:94	92:8

**Table 2:** Antimicrobial activity (mm) of the tested samples against the reference strain (a) samples and (b) controls.

Strains	(a) Samples							
	5a	5b	5c	5d		5e	5f	5g
*S. aureus*	19.96 ± 1.90	22.83 ± 1.77	22.86 ± 2.05	20.30 ± 1.05		23.03 ± 1.50	20.83 ± 0.90	22.20 ± 0.60
*E. coli*	0	13.03 ± 0.20	15.00 ± 0.26	14.50 ± 0.60		11.46 ± 0.58	13.83 ± 0.30	10.80 ± 0.40
*E. faecalis*	0	11.16 ± 0.55	11.83 ± 0.11	9.56 ± 0.23		15.46 ± 0.60	8.70 ± 0.20	9.16 ± 0.28
*K. pneumoniae*	0	8.53 ± 0.35	9.06 ± 0.15	15.33 ± 0.55		8.86 ± 0.32	16.26 ± 0.56	16.53 ± 0.58
*S. typhimurium*	0	0	0	9.33 ± 0.15		0	0	0
*C. albicans ^1^*	0	10.26 ± 0.20	11.90 ± 0.60	11.56 ± 0.92		14.53 ± 0.81	15.96 ± 0.15	10.73 ± 0.86
*C. albicans ^2^*	0	9.43 ± 0.50	13.00 ± 0.60	11.86 ± 0.81		14.06 ± 0.66	15.16 ± 0.65	10.23 ± 0.45
*C. glabrata ^3^*	0	0	8.43 ± 0.41	8.53 ± 0.45		8.83 ± 0.30	10.70 ± 0.85	0
*C. glabrata ^4^*	0	0	0	9.33 ± 0.25		0	11.13 ± 0.30	0
*C. parapsilosis*	9.53 ± 0.25	13.16 ± 2.40	16.46 ± 0.40	14.56 ± 0.06		17.43 ± 0.20	16.26 ± 0.15	11.80 ± 0.95
**Strains**	**(** **b) Controls**							
	**AMC30**	**SXT25**	**CN10**	**F300**	**AP100**	**NS100**	**KCA10**	**FLU10**
*S. aureus*	31.00 ± 0.86	27.73 ± 0.37	19.66 ± 1.35	18.16 ± 1.15	-	-	-	-
*E. coli*	19.86 ± 0.80	24.03 ± 2.70	19.40 ± 0.70	19.90 ± 0.10	-	-	-	-
*E. faecalis*	28.80 ± 0.45	26.16 ± 0.40	13.33 ± 1.13	21.53 ± 1.38	-	-	-	-
*K. pneumoniae*	16.33 ± 0.25	25.96 ± 0.30	19.60 ± 1.04	18.43 ± 1.15	-	-	-	-
*S. typhimurium*	23.53 ± 0.80	24.03 ± 1.52	15.40 ± 1.57	12.53 ± 0.75	-	-	-	-
*C. albicans ^1^*	-	-	-	-	11.63 ± 0.96	0	14.30 ± 1.70	0
*C. albicans ^2^*	-	-	-	-	11.80 ± 1.51	0	0	0
*C. glabrata ^3^*	-	-	-	-	12.33 ± 0.75	0	0	0
*C. glabrata ^4^*	-	-	-	-	16.26 ± 0.83	0	0	0
*C. parapsilosis*	-	-	-	-	12.46 ± 0.47	0	0	14.30 ± 0.26

*^1^ C. albicans* ATCC 90028, *^2^ C. albicans* ATCC 10231, *^3^ C. glabrata* ATCC 15126, *^4^ C. glabrata* ATCC 20019.

## Data Availability

Data are contained within the article.

## Supplementary Materials

The following supporting information can be downloaded at: https://www.mdpi.com/article/10.3390/antibiotics14030307/s1. Elemental analysis and copies of ^13^C-NMR spectra.
