# Supplementary material for: Antibacterial and Antifungal Properties of New Synthetic Tricyclic Flavonoids
Source: Antibiotics (Basel). 2025 Mar 16;14(3):307. doi: 10.3390/antibiotics14030307 (PMC11939415; doi:10.3390/antibiotics14030307)
Supplement: Supplementary file 1 [file antibiotics-14-00307-s001.zip › antibiotics-3476741-supplementary.pdf]

# Antibacterial and antifungal properties of new synthetic tricyclic flavonoids

**Laura Gabriela Sarbu , Irina Rosca and Mihail Lucian Birsa**

## Supplementary Material

**1. Elemental analysis** **S2**

**2. Copies of $^{13}$C NMR spectra** **S3-S16**

## 1. Elemental analysis

Elemental analyses (C, H) were conducted using a CE440 Elemental Analyser; the results were found to be in good agreement (±0.3%) with the calculated values.

**Table S1.** Elemental analysis data for the newly synthesized flavanones **4a-g** and tricyclic flavonoids **5a-g**.

| **Compound** | **% C** | | **% H** | |
|---|---|---|---|---|
| | **calcd.** | **found** | **calcd.** | **found** |
| **4a** | 54.31 | 54.53 | 4.77 | 4.61 |
| **4b** | 52.28 | 52.42 | 4.39 | 4.27 |
| **4c** | 50.56 | 50.78 | 4.24 | 4.35 |
| **4d** | 46.42 | 46.58 | 3.90 | 4.01 |
| **4e** | 55.23 | 55.51 | 5.06 | 4.92 |
| **4f** | 56.09 | 56.24 | 5.32 | 5.11 |
| **4g** | 53.44 | 53.67 | 4.89 | 5.01 |
| **5a** | 47.21 | 47.42 | 3.96 | 4.09 |
| **5b** | 45.67 | 45.49 | 3.65 | 3.35 |
| **5c** | 44.35 | 44.58 | 3.54 | 3.29 |
| **5d** | 41.14 | 41.34 | 3.29 | 3.01 |
| **5e** | 48.20 | 48.39 | 4.23 | 4.06 |
| **5f** | 49.13 | 49.34 | 4.48 | 4.28 |
| **5g** | 46.83 | 47.06 | 4.11 | 3.99 |

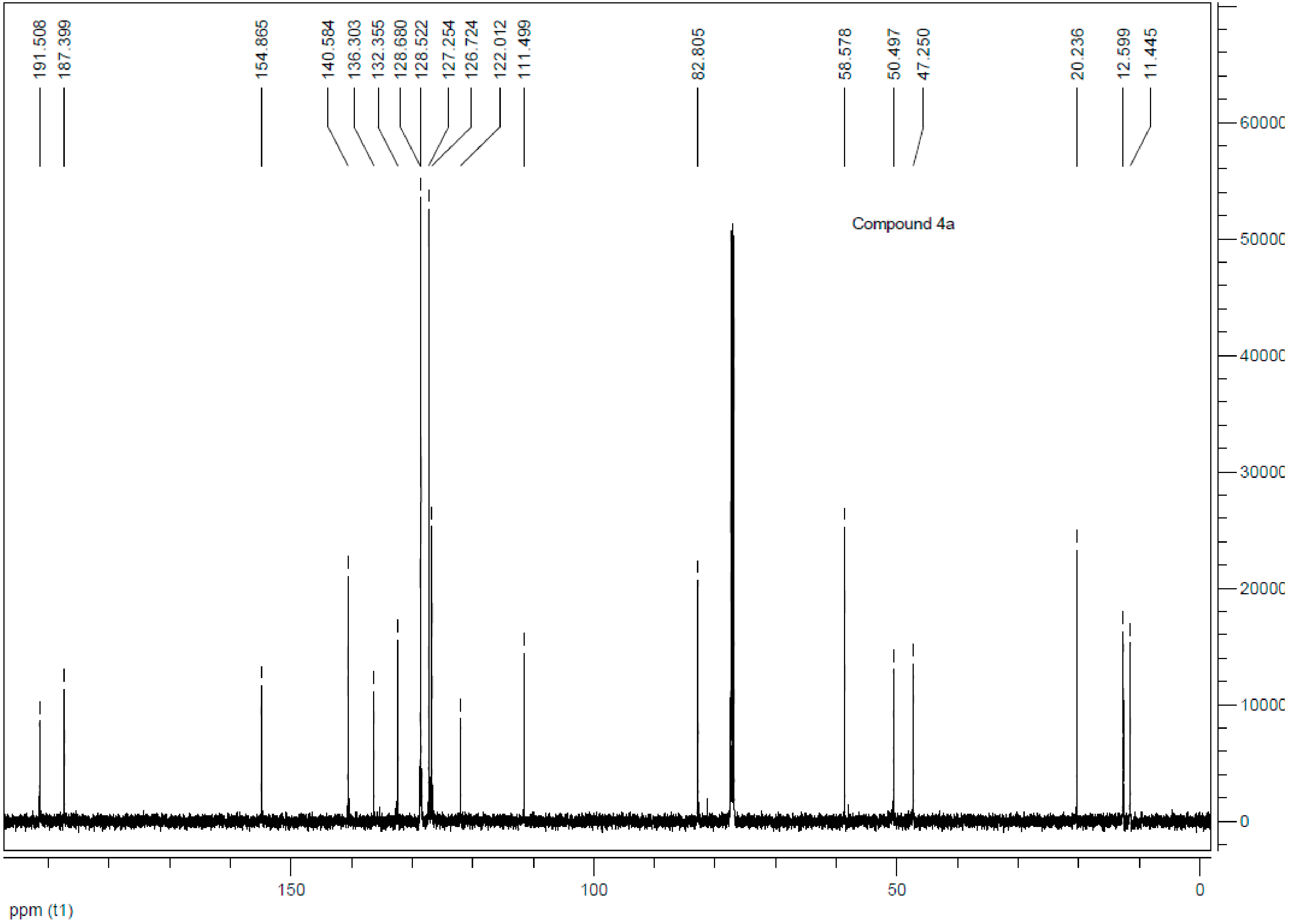
191.508
187.399
154.865
140.584
136.303
132.355
128.680
128.522
127.254
126.724
122.012
111.499
82.805
58.578
50.497
47.250
20.236
12.599
11.445
Compound 4a
60000
50000
40000
30000
20000
10000
0
150
100
50
0
ppm (t1)

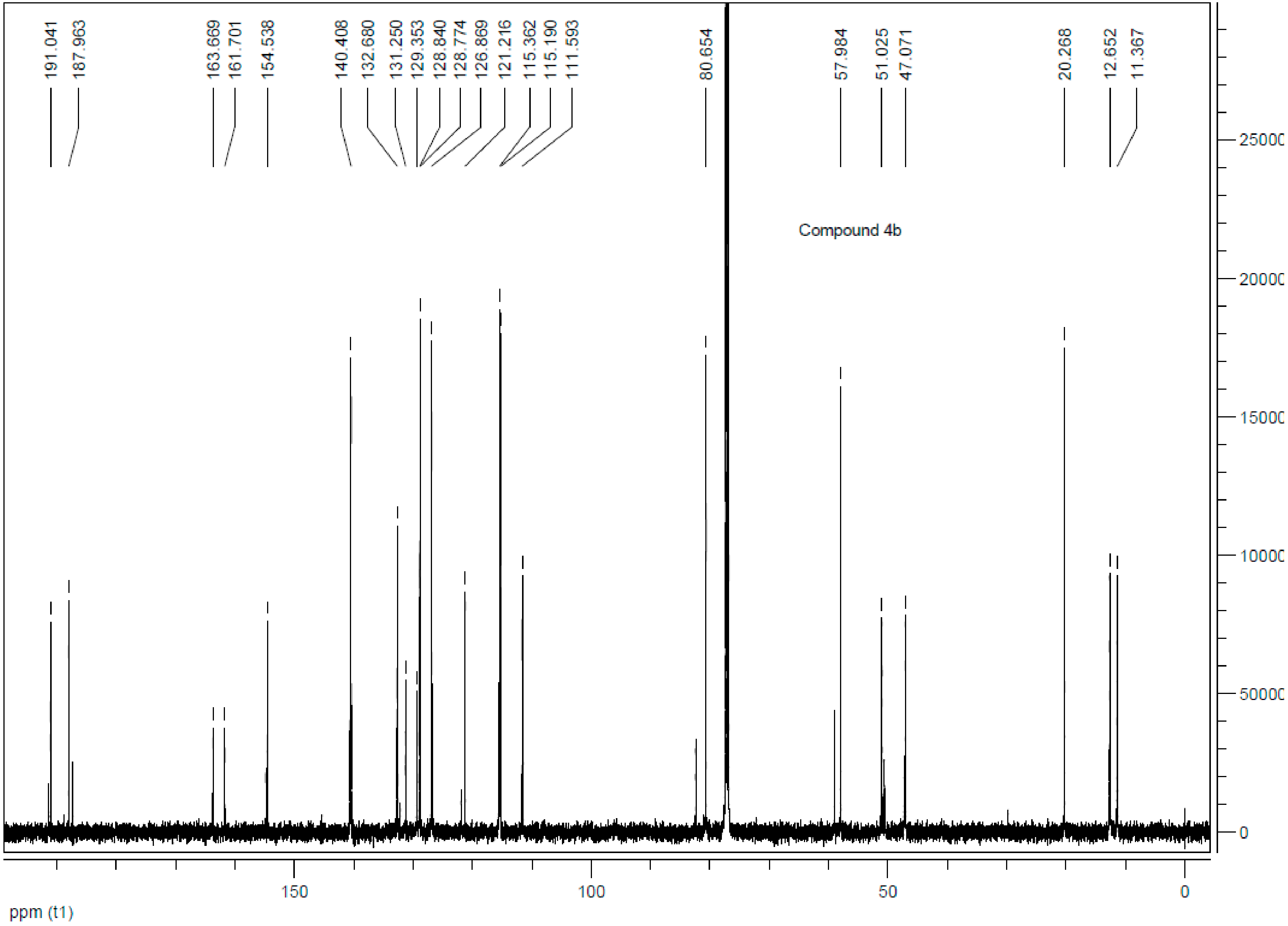

191.041
187.963
163.669
161.701
154.538
140.408
132.680
131.250
129.353
128.840
128.774
126.869
121.216
115.362
115.190
111.593
80.654
57.984
51.025
47.071
20.268
12.652
11.367
Compound 4b
25000
20000
15000
10000
50000
0
150
100
50
0
ppm (t1)

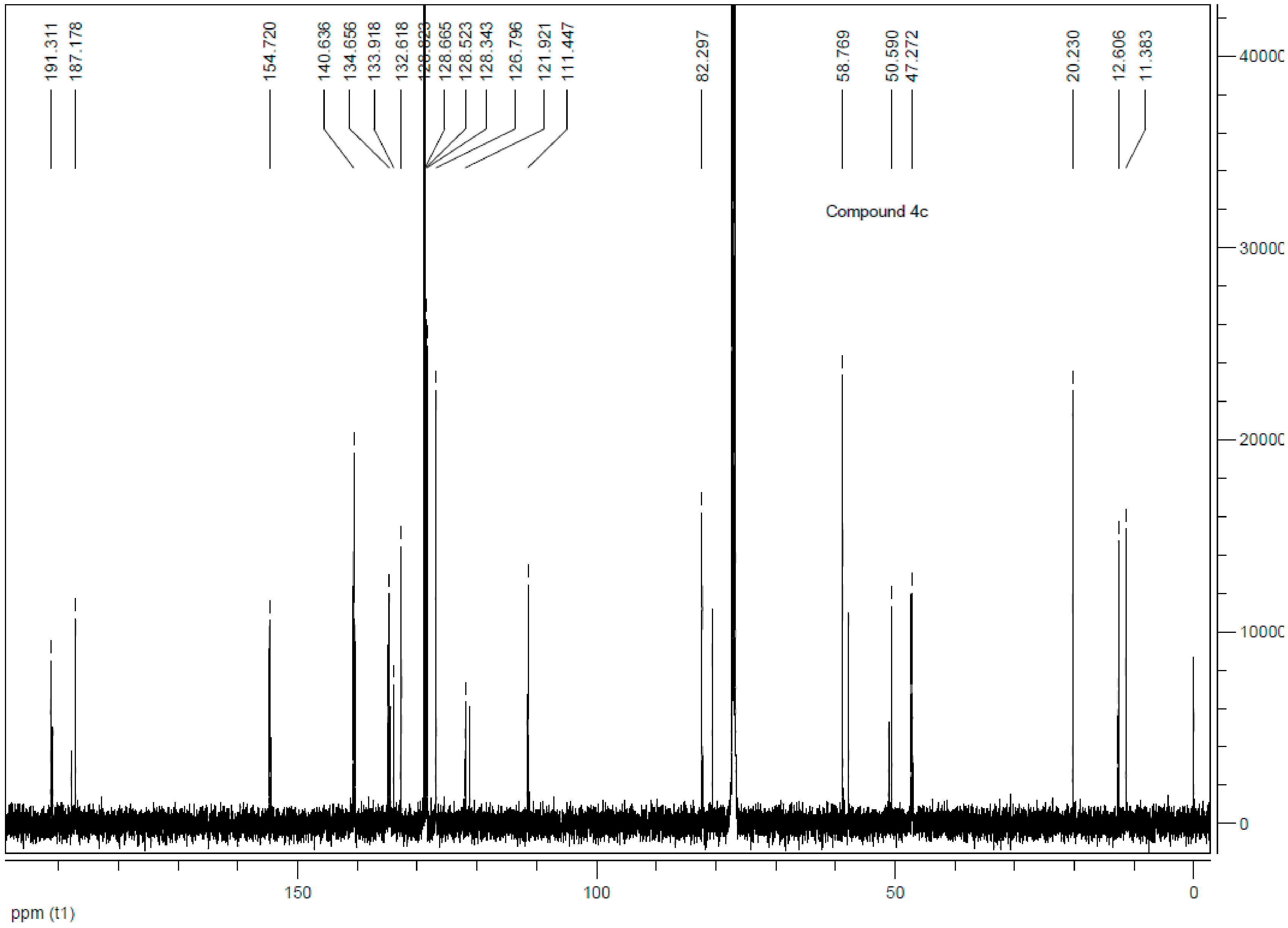

191.311
187.178
154.720
140.636
134.656
133.918
132.618
128.665
128.523
128.343
126.796
121.921
111.447
82.297
58.769
50.590
47.272
20.230
12.606
11.383
Compound 4c
150
100
50
0
ppm (t1)

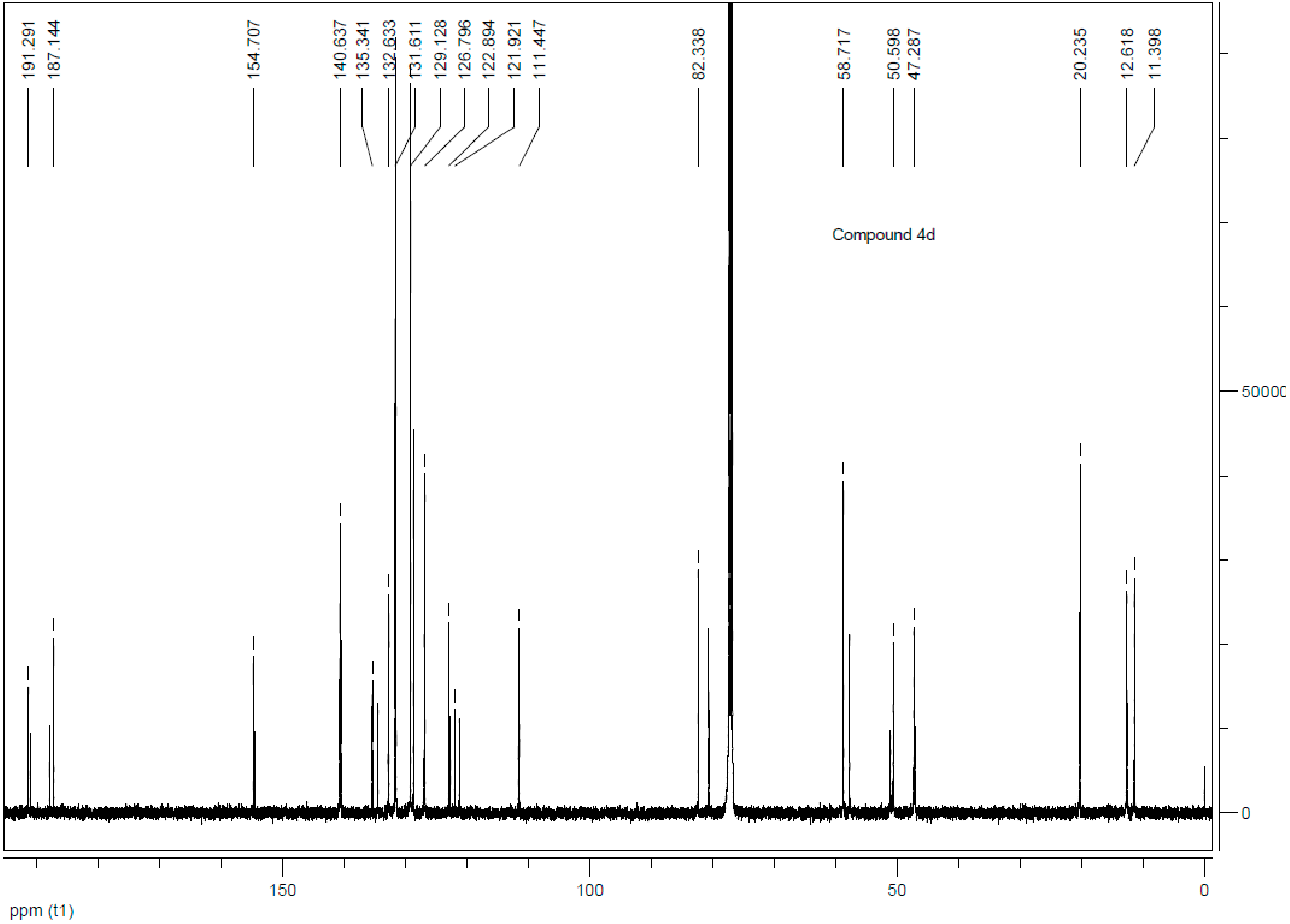

191.291
187.144
154.707
140.637
135.341
132.633
131.611
129.128
126.796
122.894
121.921
111.447
82.338
58.717
50.598
47.287
20.235
12.618
11.398
Compound 4d
50000
0
150
100
50
0
ppm (t1)

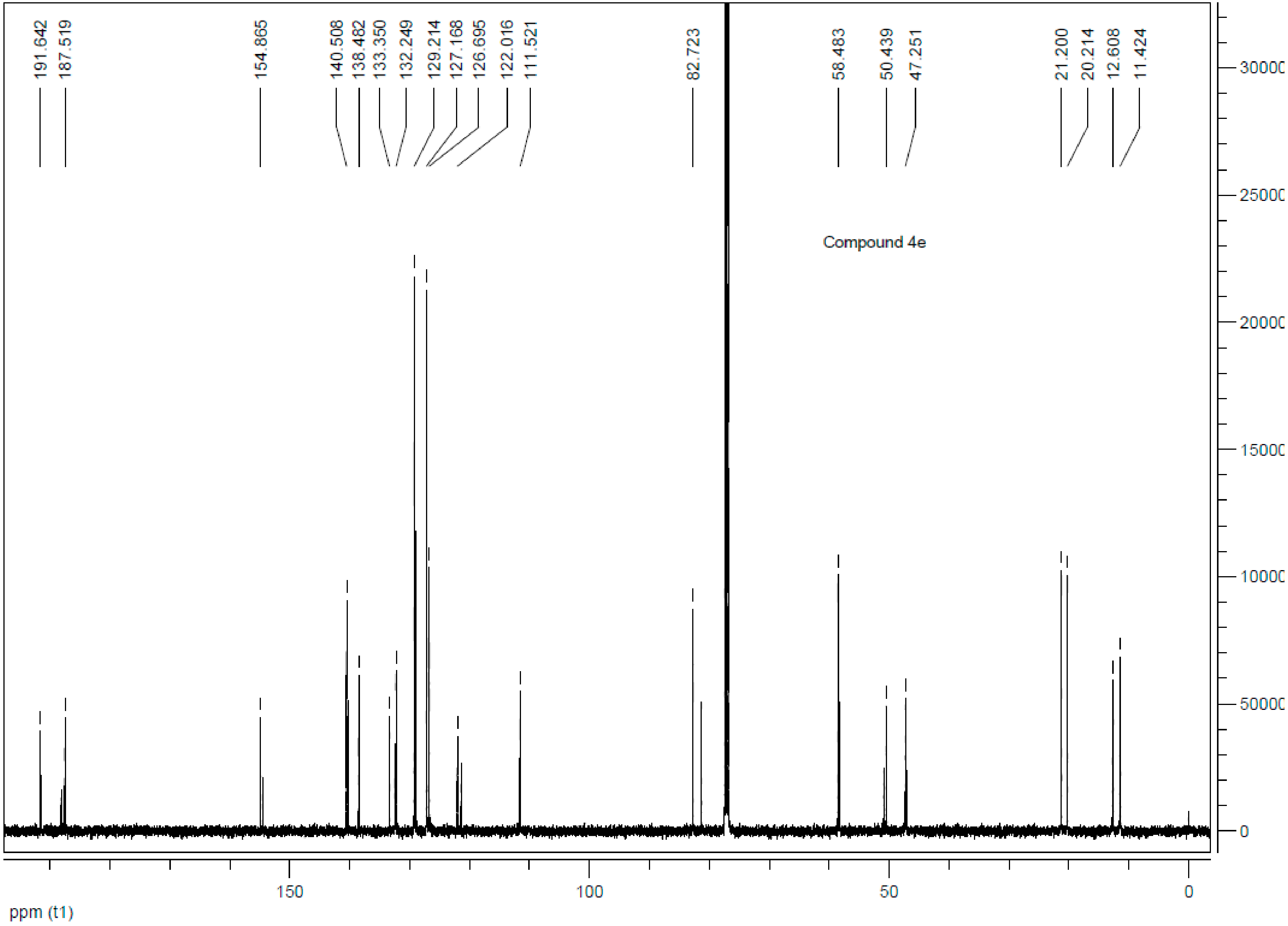
191.642
187.519
154.865
140.508
138.482
133.350
132.249
129.214
127.168
126.695
122.016
111.521
82.723
58.483
50.439
47.251
21.200
20.214
12.608
11.424
Compound 4e
ppm (t1)
150
100
50
0

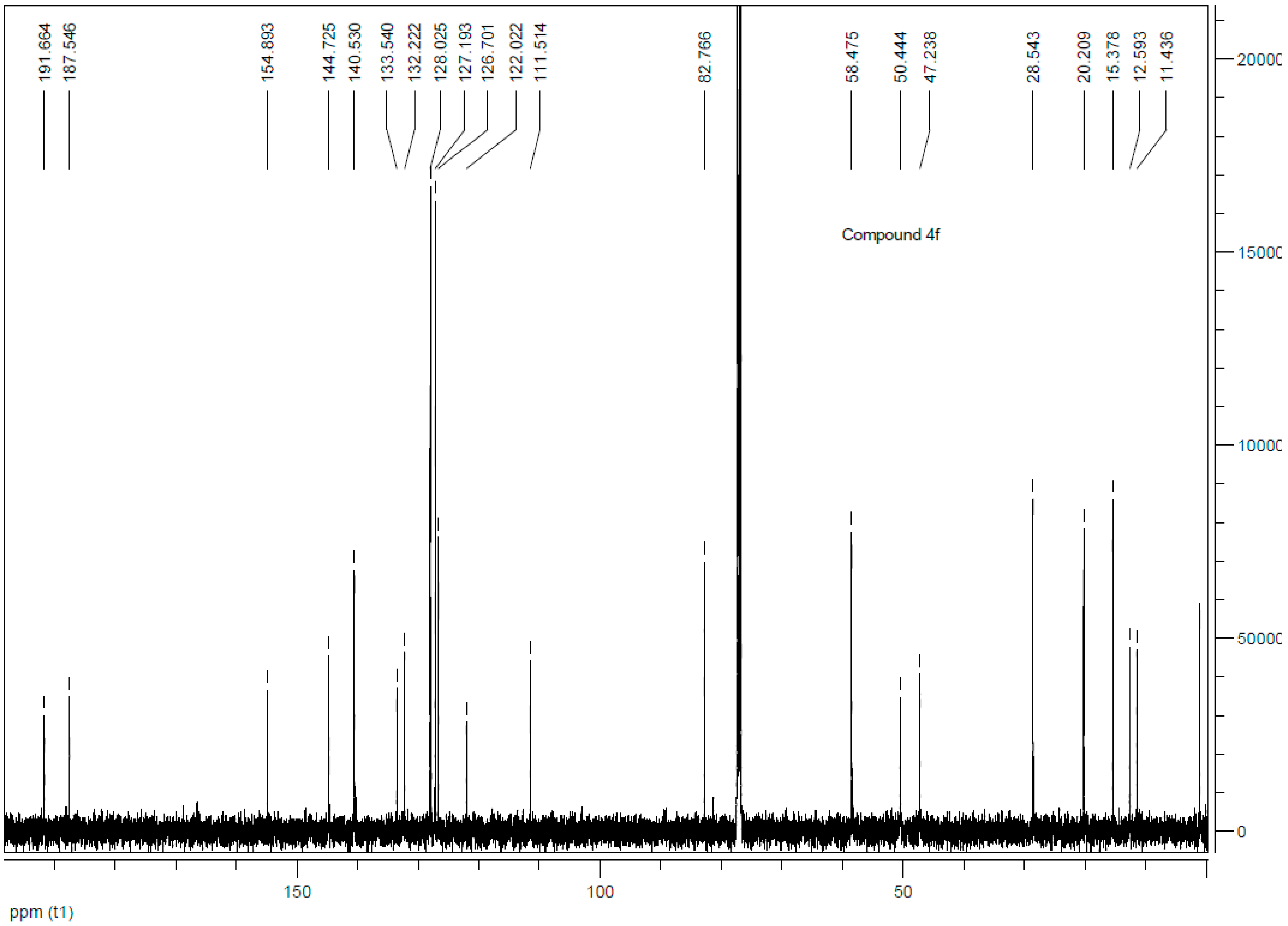
191.664
187.546
154.893
144.725
140.530
133.540
132.222
128.025
127.193
126.701
122.022
111.514
82.766
58.475
50.444
47.238
28.543
20.209
15.378
12.593
11.436
Compound 4f
ppm (t1)
150
100
50
0

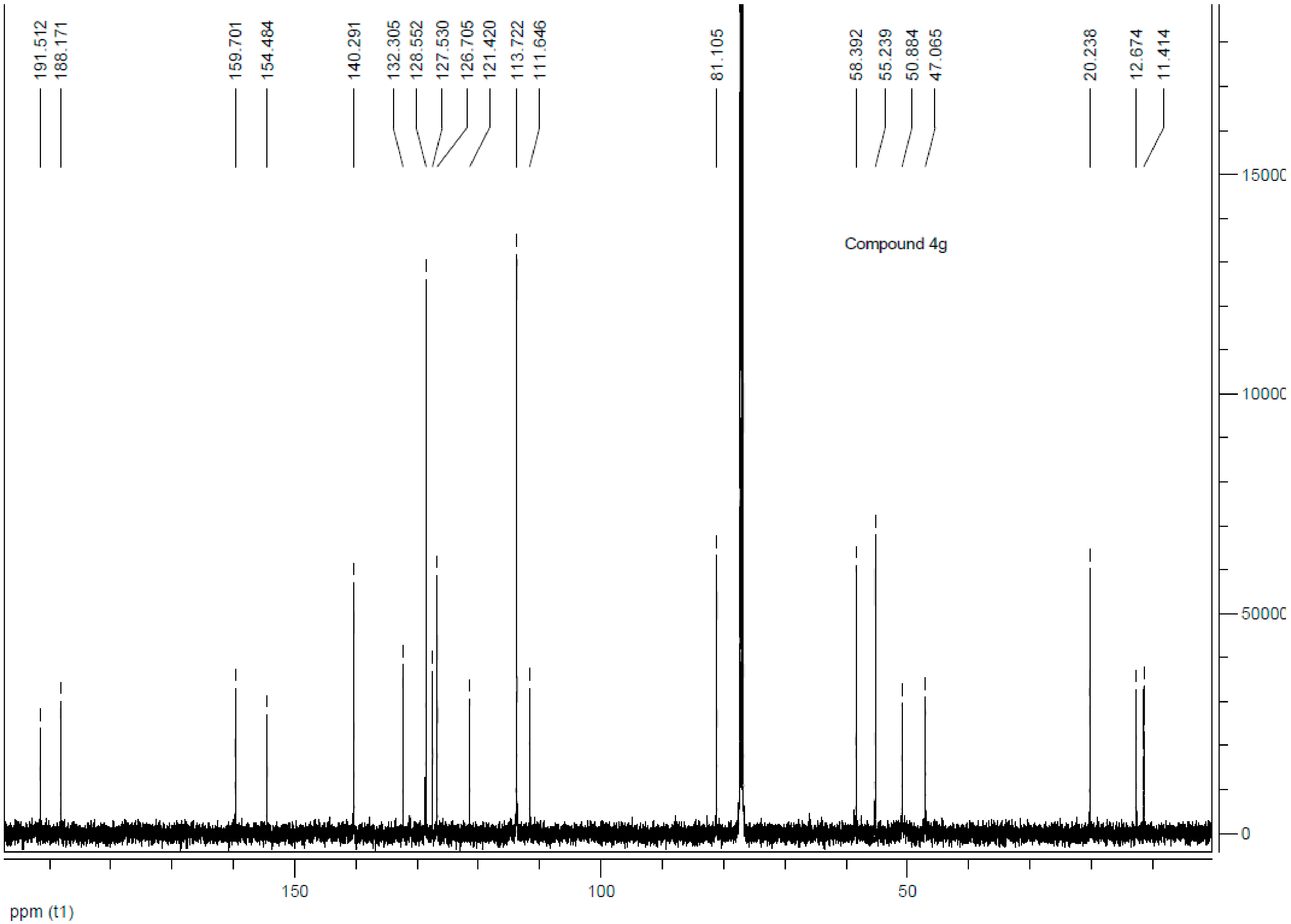
191.512
188.171
159.701
154.484
140.291
132.305
128.552
127.530
126.705
121.420
113.722
111.646
81.105
58.392
55.239
50.884
47.065
20.238
12.674
11.414
Compound 4g
150
100
50
ppm (t1)
15000
10000
50000
0

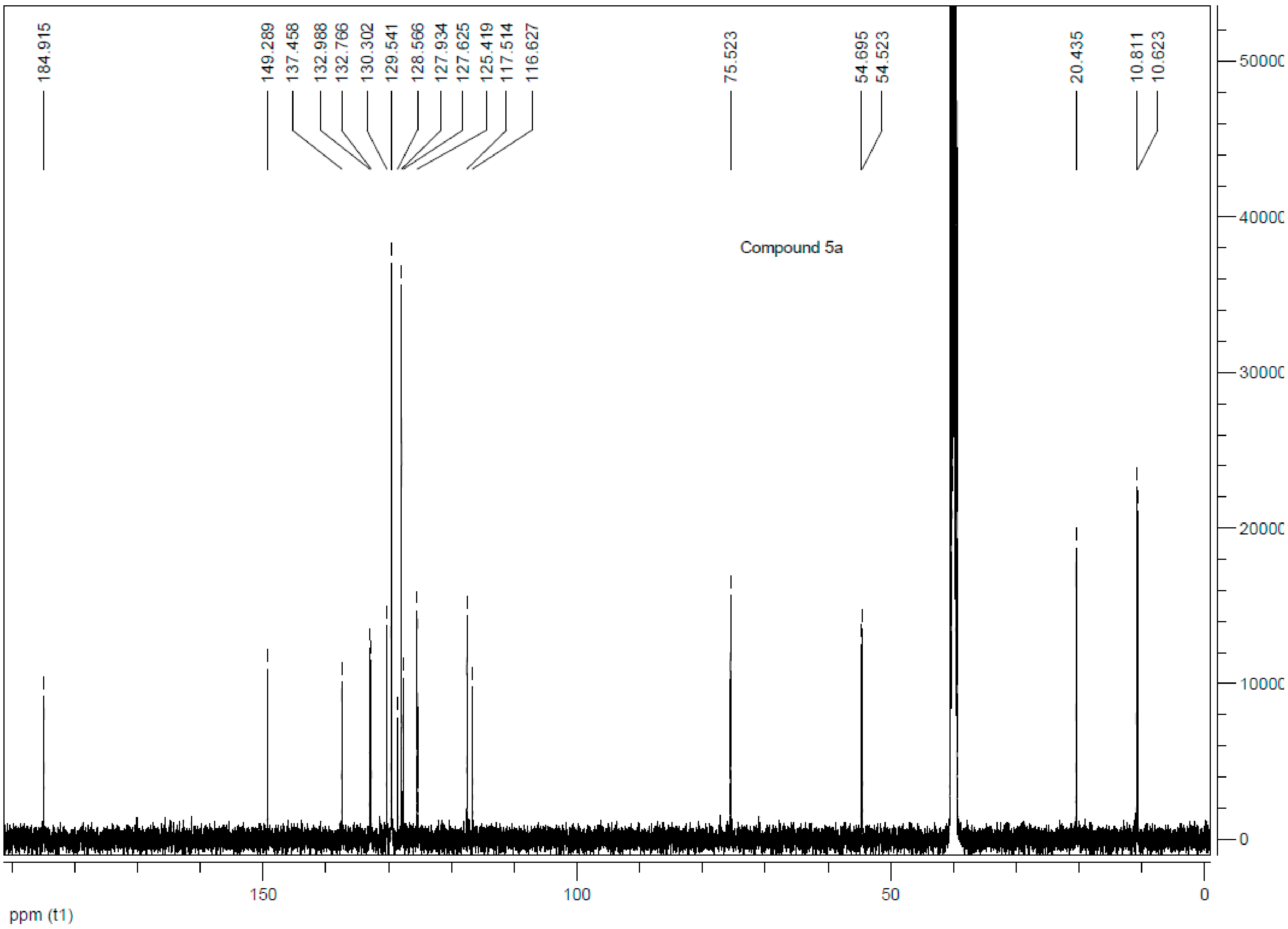
184.915
149.289
137.458
132.988
132.766
130.302
129.541
128.566
127.934
127.625
125.419
117.514
116.627
75.523
54.695
54.523
20.435
10.811
10.623
Compound 5a
50000
40000
30000
20000
10000
0
150
100
50
0
ppm (t1)

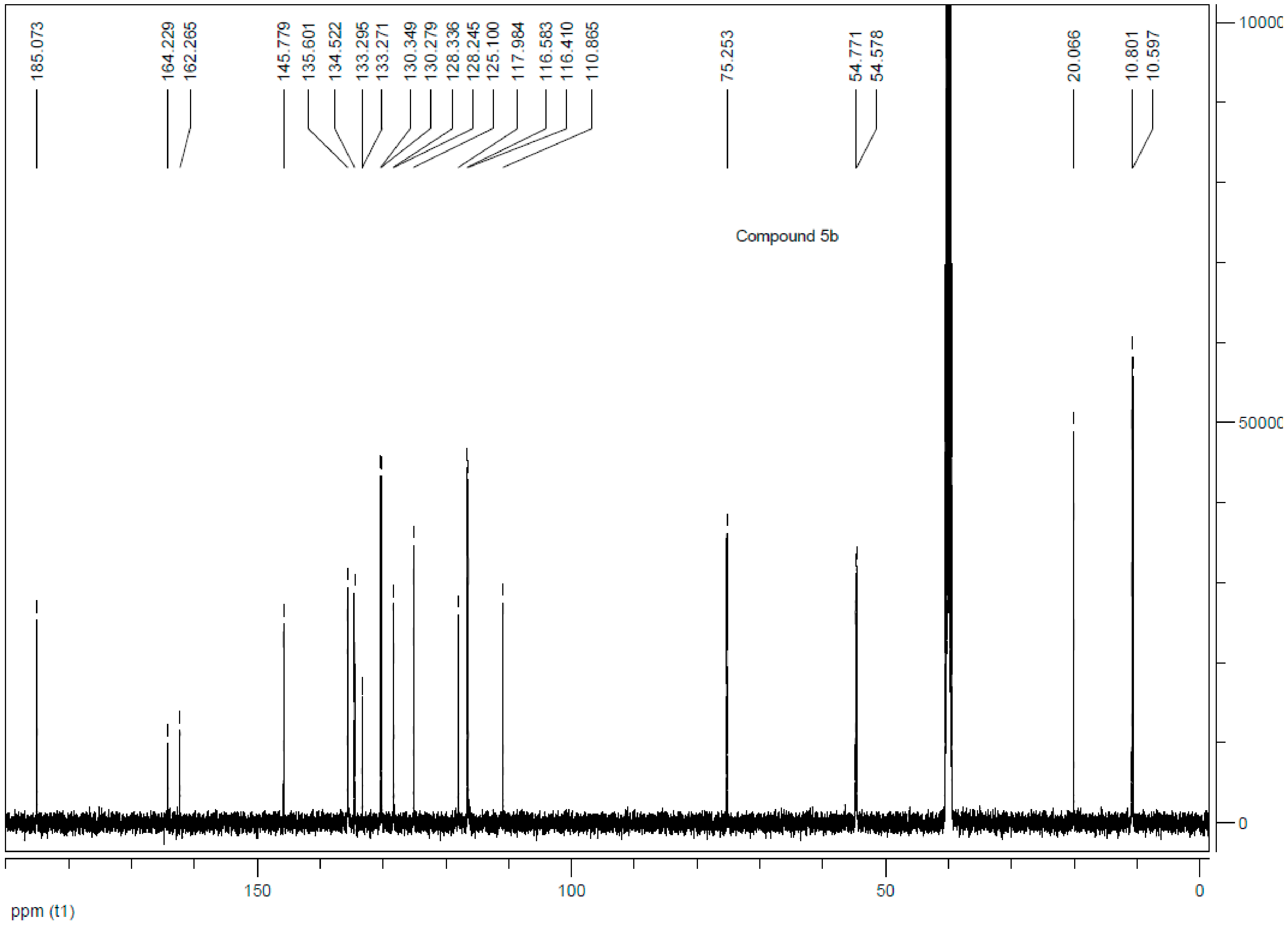

185.073
164.229
162.265
145.779
135.601
134.522
133.295
133.271
130.349
130.279
128.336
128.245
125.100
117.984
116.583
116.410
110.865
75.253
54.771
54.578
20.066
10.801
10.597
Compound 5b
10000
50000
0
150
100
50
0
ppm (t1)

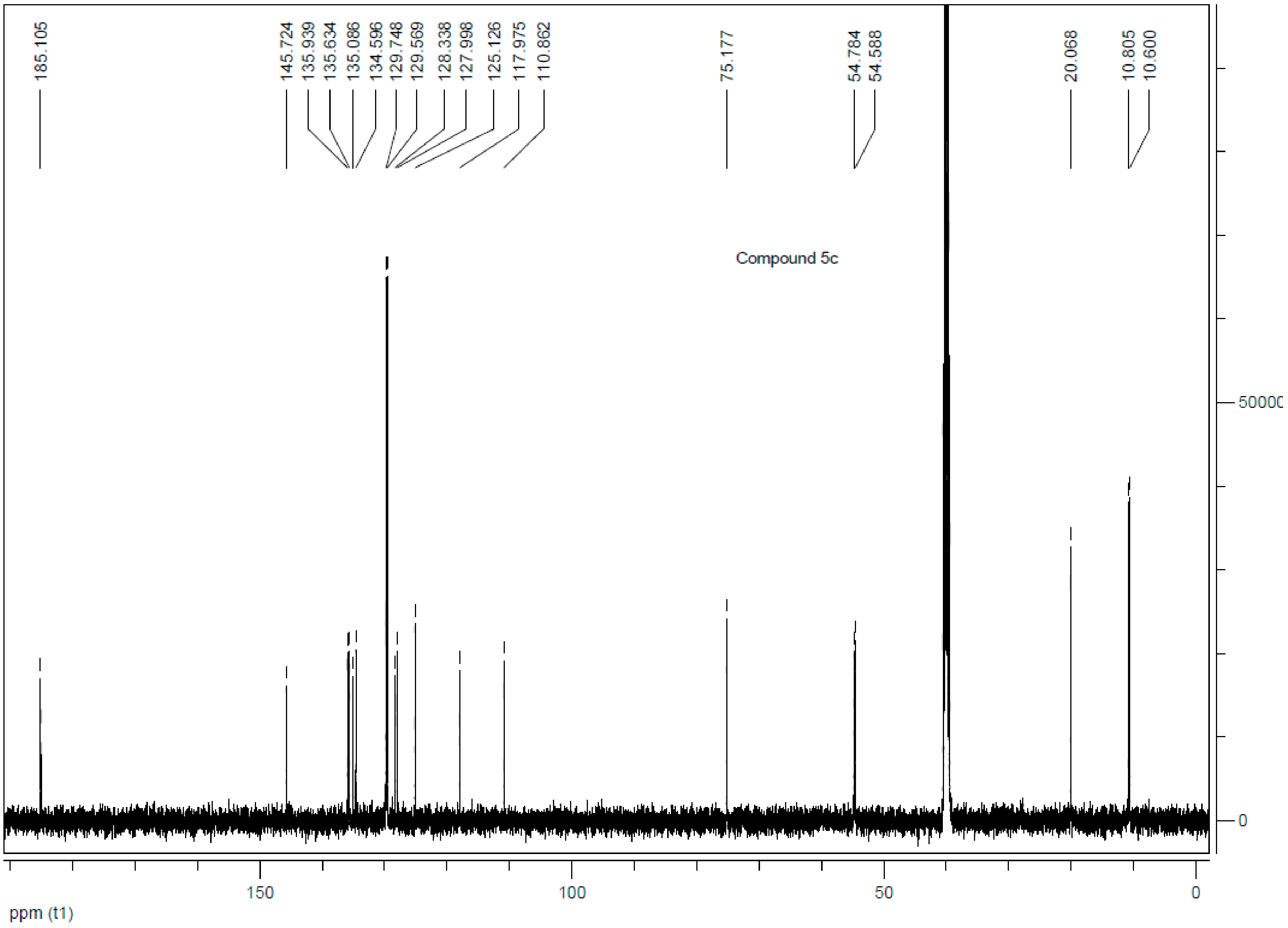
185.105
145.724
135.939
135.634
135.086
134.596
129.748
129.569
128.338
127.998
125.126
117.975
110.862
75.177
54.784
54.588
20.068
10.805
10.600
Compound 5c
50000
0
150
100
50
0
ppm (t1)

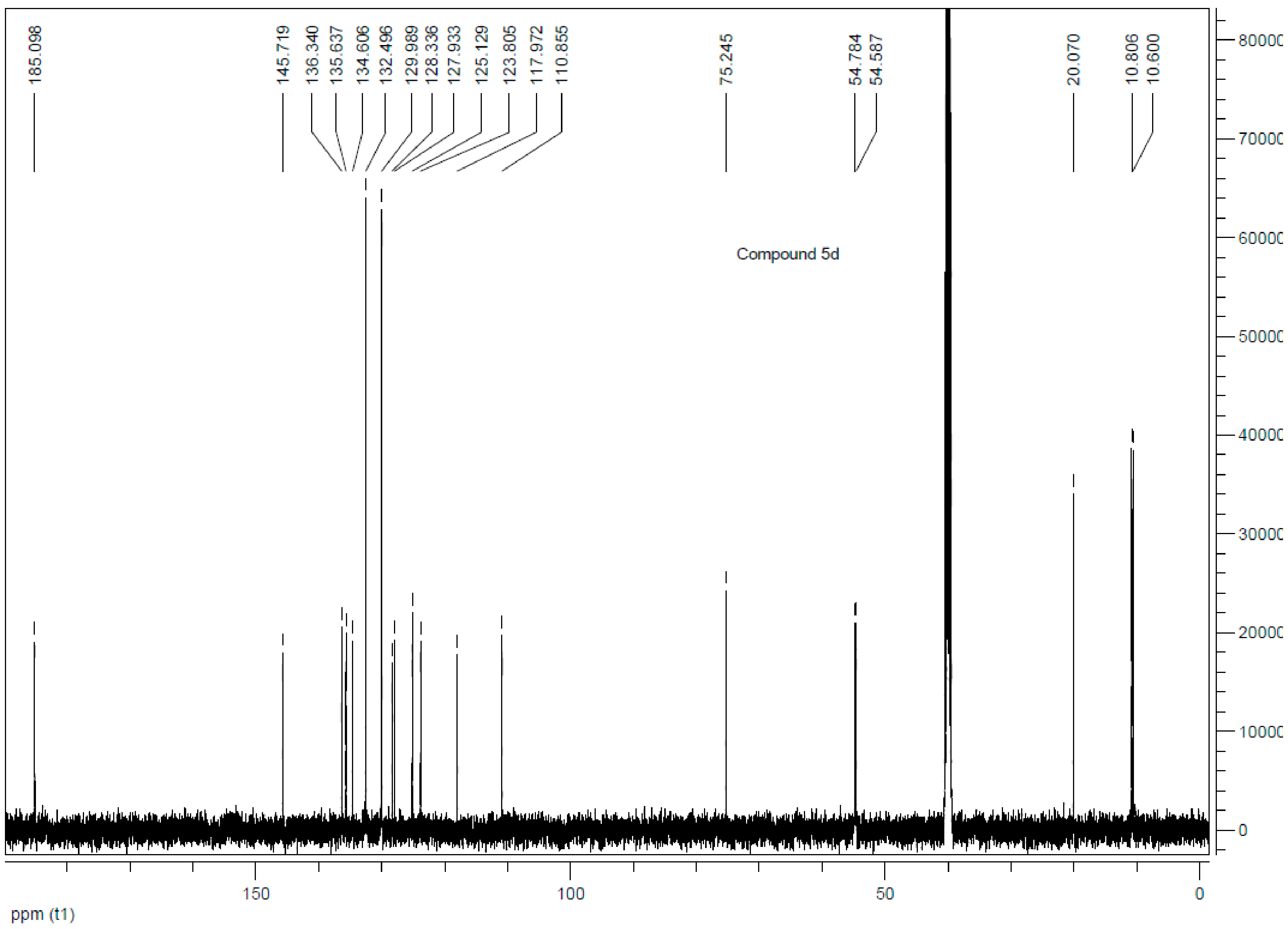

185.098
145.719
136.340
135.637
134.606
132.496
129.989
128.336
127.933
125.129
123.805
117.972
110.855
75.245
54.784
54.587
20.070
10.806
10.600
Compound 5d
ppm (t1)

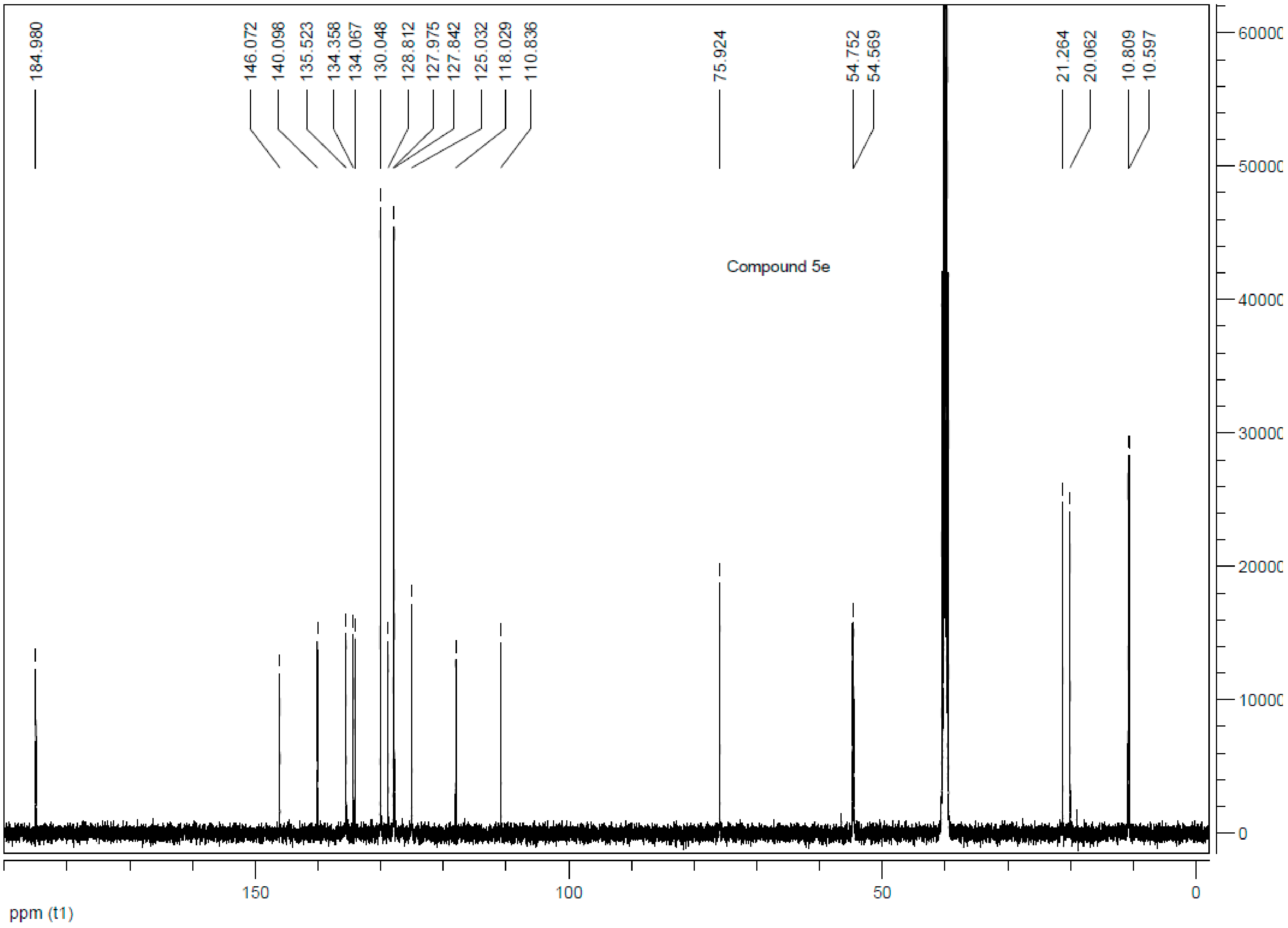
184.980
146.072
140.098
135.523
134.358
134.067
130.048
128.812
127.975
127.842
125.032
118.029
110.836
75.924
54.752
54.569
21.264
20.062
10.809
10.597
Compound 5e
ppm (t1)

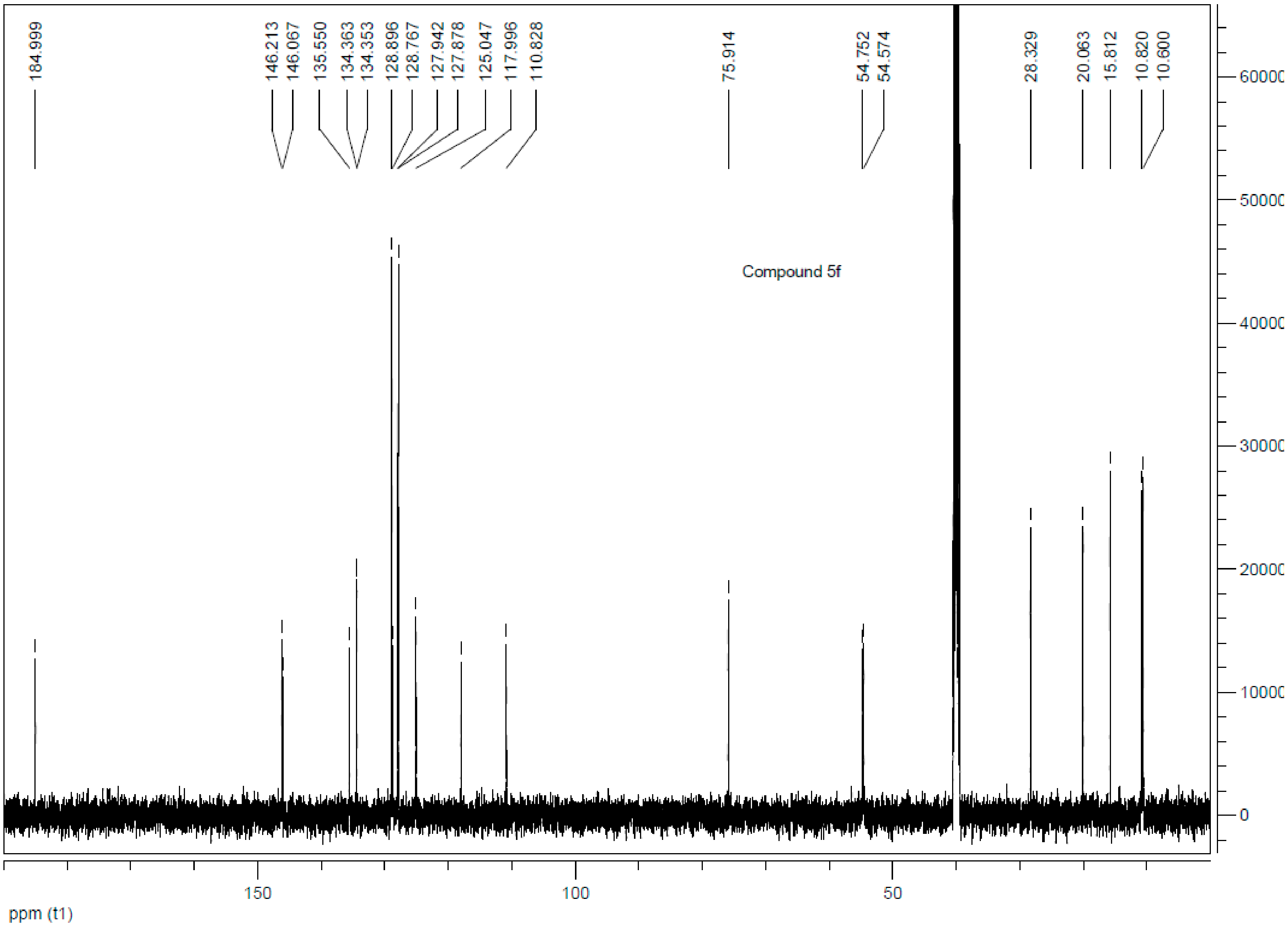
184.999
146.213
146.067
135.550
134.363
134.353
128.896
128.767
127.942
127.878
125.047
117.996
110.828
75.914
54.752
54.574
28.329
20.063
15.812
10.820
10.600
Compound 5f
60000
50000
40000
30000
20000
10000
0
150
100
50
ppm (t1)

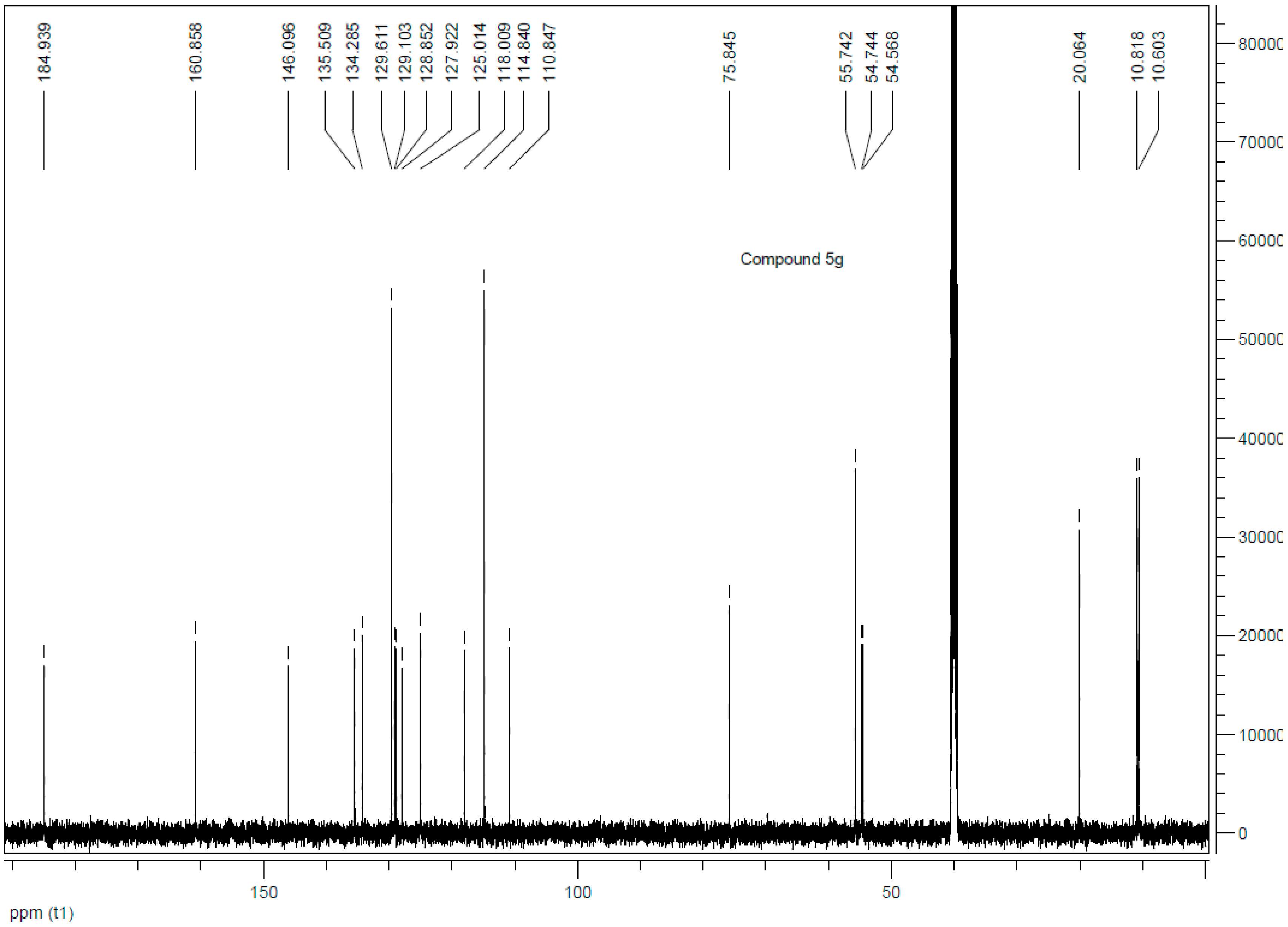
184.939
160.858
146.096
135.509
134.285
129.611
129.103
128.852
127.922
125.014
118.009
114.840
110.847
75.845
55.742
54.744
54.568
20.064
10.818
10.603
Compound 5g
80000
70000
60000
50000
40000
30000
20000
10000
0
150
100
50
ppm (t1)
